# Enhancing Patient and Staff Engagement in the Transition from Monthly to 2-Monthly Aripiprazole Long-Acting Injectable Therapy: A Quality Improvement Project

**DOI:** 10.1192/bjo.2026.11457

**Published:** 2026-06-30

**Authors:** Nora Paul, Surabhi Hullumane

**Affiliations:** Black Country Healthcare NHS Foundation Trust, West Bromwich, United Kingdom

## Abstract

**Aims:**

To identify patient- and staff-reported barriers and facilitators influencing transition from monthly to 2-monthly aripiprazole long-acting injectable (LAI) therapy within an Adult Mental Health Outpatient Service, and to use these findings to design interventions to improve the consistency and quality of patient–clinician discussions.

**Methods:**

Structured, anonymous surveys were administered to patients receiving monthly Aripiprazole LAI (n=11) and multidisciplinary staff involved in LAI prescribing or administration (n=23). Surveys included Likert-scale items and free-text questions exploring understanding, willingness, confidence, perceived barriers, and facilitators. Quantitative data were analysed descriptively, and free-text responses reviewed thematically. Findings informed staged interventions tested through Plan–Do–Study–Act (PDSA) cycles.

**Results:**

Patients: Most had long-term monthly LAI exposure (55% >5 years). Understanding of differences between monthly and 2-monthly LAI was limited (45% reporting “not at all” or “a little”). Willingness to switch was polarised: 45% not willing, while 45% willing or very willing. Key concerns included fear of relapse (18%), concern that the injection may not remain effective for two months (27%), fewer clinical contacts (27%), and prolonged side effects (27%). Perceived benefits included fewer injections (73%), increased convenience/flexibility (55%), and fewer clinic visits or reduced travel (45% each). Over half (55%) requested more information, and 36% reported greater willingness to switch if better informed.

Staff: Although 87% of staff were aware of the 2-monthly formulation, 43% had never discussed it with patients. While 52% felt confident discussing the option, only 26% felt confident with clinical switching considerations, and 43% reported low clarity regarding practical steps such as the Named Patient Registration Form. Common barriers included patient resistance (35%), fear of relapse or destabilisation (35%), concern regarding sustained effectiveness (30%), reduced clinical monitoring (26%), and lack of patient education materials (35%). Facilitators included patient leaflets (78%), structured guidance or checklists (57%), clear explanation of benefits and risks (>55%), and staff training or induction (70%).

**Conclusion:**

Baseline patient and staff data demonstrate that transitions to 2-monthly aripiprazole LAI are limited not by lack of awareness, but by gaps in patient understanding, clinician confidence with clinical and practical switching considerations, and absence of consistent education and process tools. The co-designed intervention–comprising a communication protocol, staff checklist, patient leaflet, targeted staff training, and peer-supported patient education–targets these modifiable gaps. This QIP highlights the value of aligning patient and staff perspectives to strengthen shared decision-making and improve consistency in LAI care, with implications for future service-level optimisation.

